# Phenolic Composition and Antioxidant Capacity in Table Grape Berries Following Natural Hail Damage

**DOI:** 10.3390/ijms27125284

**Published:** 2026-06-11

**Authors:** Despoina G. Petoumenou, Ioannis Daskalakis, Katerina Biniari

**Affiliations:** 1 Laboratory of Viticulture, Department of Agriculture Crop Production and Rural Environment, University of Thessaly, Fytokou Street, 38446 Volos, Greece; 2 Laboratory of Viticulture, Department of Crop Science, Agricultural University of Athens, Iera Odos, 75, 11855 Athens, Greece

**Keywords:** abiotic stress, climate change, *Vitis vinifera* L., phenolic compounds, antioxidant activity, proline, DPPH, resveratrol, flavonols, flavanols, non-flavonoids, PCA

## Abstract

Phenolic compounds, essential for grape quality, are affected by environmental factors, including abiotic stressors such as hail. This study examined the impact of varying levels of natural hail damage on the physicochemical parameters, phenolic composition, and antioxidant capacity of Thompson seedless grapevines (*Vitis vinifera* L.) under Mediterranean climatic conditions. The research was carried out in a commercial vineyard in Greece during the 2015 growing season, following a major hailstorm. Three treatments were implemented: control (undamaged), moderate hail damage, and total hail damage. The results showed that pH levels and specific physiological parameters, including proline concentration, were significantly influenced across treatments. Detailed analysis revealed that the concentrations of phenolic compounds generally increased with greater hail damage, indicating enhanced antioxidant capacity and metabolic adaptation to natural hail-induced mechanical stress. Additionally, individual phenolic compounds, such as flavanols, hydroxybenzoic acids, and stilbenes, responded differently to hail damage, demonstrating complex regulatory mechanisms in grape metabolism. These findings underscore the importance of understanding grapevine biochemical responses to extreme weather events in the context of climate change, as changes in phenolic composition can directly affect grape quality.

## 1. Introduction

Grapevines (*Vitis vinifera* L.) are perennial woody plants well adapted to Mediterranean climates, and the Thompson seedless cultivar is among the most widely cultivated table-grape varieties in Greece [1]. However, viticulture faces increasing challenges from the frequency of extreme weather events associated with climate change [2]. Among these weather phenomena, hailstorms can cause significant damage to grapevines through mechanical injury. A previous study showed that natural hail significantly reduced the total leaf area, yield, and total phenolic compounds in the berries of Thompson seedless [3]. The degree of injury largely depends on factors such as hailstone size and storm duration.

Grape quality is determined by a complex interaction of morphological characteristics, primary metabolites (sugars and organic acids), and secondary metabolites, with phenolic compounds being the most important. These phenolic compounds, such as flavonoids, phenolic acids, tannins, and stilbenes, play a crucial role in the organoleptic profile of grapes and their overall quality, contributing to bitterness and astringency, while simultaneously providing significant antioxidant capacity [4,5]. In grapes, phenolic compounds are broadly classified into two major groups: non-flavonoids (including hydroxybenzoic acids, hydroxycinnamic acids, and stilbenes) and flavonoids (such as flavones, flavonols, flavanones, flavan-3-ols, and anthocyanins) [6], with their accumulation strongly influenced by environmental conditions and cultivation practices. These compounds are also essential in plant defence, contributing to tolerance against both biotic and abiotic stresses, including high irradiance, low temperatures, drought, and heavy-metal exposure. Stress conditions frequently stimulate phenolic accumulation, thereby mitigating deleterious effects [7]. This response is mediated through stress-induced cell signalling that leads to the transcriptional up-regulation of the phenylpropanoid pathway [8], resulting in increased biosynthesis of phenolic compounds [6,9,10]. These compounds serve not only as quality indicators but also as integral components of the plant’s defence system, protecting tissues from oxidative damage.

Hail events constitute a form of mechanical abiotic stress in viticultural systems, inducing physical injury to vegetative and reproductive tissues. Depending on intensity and grapevine phenological stage, hail can inflict injuries upon leaves, shoots, inflorescences, canes, and berries, thereby compromising vegetative growth, yield, and fruit quality [2] and, in severe cases, vineyard survival. Beyond overt physical damage, mechanical injury can elicit significant metabolic adaptations in berry skin tissues. Such injuries have been associated with oxidative stress, membrane integrity disruption, and activation of stress-related signalling pathways, which subsequently stimulate phenolic compound and antioxidant molecule accumulation [11,12]. Although the effects of abiotic stress—such as water deficit, extreme temperatures, and high irradiance—on grapevine phenolic metabolism have been extensively investigated, the biochemical response of grapevines to natural hail remains poorly characterized.

Phenolic metabolism in *Vitis vinifera* L. is particularly susceptible to environmental perturbations and plays a central role in stress adaptation [8]. Flavanols (catechin, epicatechin, and procyanidins), flavonols (quercetin and rutin), hydroxybenzoic and hydroxycinnamic acids, as well as stilbenes—notably resveratrol and ε-viniferin—are known to accumulate under stress conditions, thereby contributing to enhanced antioxidant capacity [13,14]. Concurrently, amino acids—notably proline—frequently accumulate under abiotic stress, participating in the regulation of osmotic balance and redox status [15,16]. Therefore, the integrated evaluation of maturation indices, phenolic composition, and antioxidant capacity can provide substantial insight into the physiological adaptation of berries to mechanical stress induced by hail damage.

In the context of contemporary climate instability, as extreme weather events become increasingly frequent and intense, investigation of natural hail effects on grape metabolism is of particular significance. Understanding metabolic adaptations is crucial not only for plant physiology but also for grape quality management, given that alterations in phenolic composition can significantly affect the organoleptic and functional characteristics of grapes and derived products. The aim of this study was to investigate the effects of varying levels of natural hail damage (control, moderate, and total) on must physicochemical parameters, grape skin phenolic composition, and antioxidant capacity. Particular emphasis was placed on the determination of individual phenolic compounds by High Performance Liquid Chromatography (HPLC) analysis, as well as on elucidating correlations and performing principal component analysis (PCA) to clarify coordinated metabolic responses of grapevine berries under stress induced by natural hail.

## 2. Results

### 2.1. Effect of Natural Hail Damage on Total Soluble Solids (TSS), pH, Titratable Acidity (TA), Proline, and Arginine

The results presented in Table 1 show that the total soluble solids (°Brix) were not statistically significantly affected by the treatments, despite an apparent increase under total damage conditions. Similarly, titratable acidity and ripening index exhibited no statistically significant differences among damage levels, suggesting that hail did not substantially alter the general sugar–acid balance of the must. In contrast, pH differed significantly among treatments, with total damage resulting in elevated values compared to the control, whereas moderate damage yielded intermediate values that did not differ significantly from either extreme. Of particular interest is the amino acid profile of the must. Proline was significantly affected by hail damage, exhibiting the lowest concentration under moderate damage and the highest under total damage, likely reflecting physiological stress responses within the berries. Conversely, arginine concentrations did not differ statistically between treatments. Collectively, these findings indicate that, although hail did not significantly affect key ripening indices, it induced alterations in specific physiological parameters—notably pH and proline content—reflecting an adaptive response to severe mechanical injury.

### 2.2. Effect of Natural Hail Damage on Phenolic Profile and Antioxidant Activity

A clear trend of increasing phenolic compound concentrations was observed with escalating damage intensity, with maximal values recorded under total hail damage conditions (Table 2). Flavanols in grape skins exhibited a significant increase in both moderate and total damage treatments compared to the control; however, no significant differences were detected between the two damage levels. A similar pattern was observed for total tannins (total condensed tannins), wherein the highest concentration was recorded under total damage conditions, differing significantly from the control, whereas moderate damage yielded intermediate values. For flavonoids, a progressive and statistically significant increase was observed with increasing hail intensity, with maximal accumulation under total damage. Similarly, flavones, flavonols, and total phenolics attained significantly higher concentrations under total damage compared to the control, whereas moderate damage yielded intermediate values. Antioxidant capacity, as assessed by Ferric Reducing Antioxidant Power (FRAP) and 2,2-Diphenyl-1-picrylhydrazyl (DPPH) free radical scavenging assays, increased significantly with damage intensity, exhibiting the lowest values in the control and the highest under total hail damage. These findings indicate an enhancement of grape defence mechanisms through elevated synthesis of phenolic compounds in response to hail-induced stress. Collectively, the results suggest that hail, particularly at high intensity, activates metabolic processes leading to phenolic compound accumulation and increased antioxidant activity in grape skins—a response of considerable significance for both plant physiology and grape quality characteristics.

### 2.3. Effect of Natural Hail Damage on Individual Flavanols

Individual flavanols increased with hail damage intensity, with maximal values recorded under total hail damage conditions (Table 3). Specifically, catechin exhibited progressive elevation from control through moderate to total damage, with all treatments differing significantly. A similar trend was observed for epicatechin, wherein total hail damage resulted in a significantly higher concentration compared to other treatments. Regarding procyanidins, procyanidin B1 exhibited significantly higher values under total damage compared to control and moderate damage, which did not differ significantly. Conversely, procyanidin B2 showed a statistically significant increase corresponding to hail intensity, with the highest concentration measured under total damage. Overall, these findings suggest that hailstorms activate flavanol biosynthesis in grape skins, possibly as a defence mechanism against mechanical injury and subsequent oxidative stress. Furthermore, the significant increase in these compounds—which are key contributors to bitterness and astringency—may substantially affect the organoleptic profile and the overall antioxidant characteristics of grapes. Such responses are of particular importance for understanding both plant physiological resilience and the resulting grape quality characteristics.

### 2.4. Effect of Natural Hail Damage on Hydroxybenzoic Acid Profiles

Table 4 presents the concentration of hydroxybenzoic acids in berry skins subjected to natural hail damage. The results indicate that hail significantly affected most of the phenolic acids examined, with differences corresponding to damage intensity. Protocatechuic acid exhibited significantly higher concentrations under both moderate and total damage compared to the control, with no statistically significant difference between damage levels. In contrast, gallic acid reached its highest concentration under total damage, which differed significantly from both control and moderate damage, whereas the latter two were statistically comparable. Vanillic acid also showed a significant increase under total hail damage, while control and moderate damage recorded lower and statistically similar values. For syringic acid, significant differences were observed among all treatments, with moderate damage exhibiting the highest concentration, followed by total damage and subsequently the control. Overall, these findings suggest that hailstorms alter hydroxybenzoic acid profiles in berry skins. The enhanced accumulation of these compounds likely presents a metabolic adaptation to fortify cell wall structures and increase precursor availability for synthesis of more complex protective polyphenols. The differential response among individual acids indicates complex regulation of phenolic metabolism under varying degrees of mechanical injury.

### 2.5. Effect of Natural Hail Damage on Hydroxycinnamic Acids and Their Respective Tartaric Acids Esters

The concentrations of hydroxycinnamic acids and their respective tartaric acid esters in berry skins in relation to the extent of natural hail damage are summarized in Table 5. Notably, sinapic acid showed a marked, progressive decline with increasing damage intensity, with the highest concentration in control berry skins and the lowest under total hail damage. Conversely, caffeic acid increased significantly with hail intensity, reaching peak values under total damage conditions. In contrast, *p*-coumaric acid showed no significant differences among treatments, although a decreasing trend was observed with increasing damage intensity. Meanwhile, *m*-coumaric acid exhibited significantly higher concentrations under both moderate and total hail damage compared to the control, with no significant difference between the two damage levels. Regarding esterified derivatives, caftaric and fertaric acids increased significantly with damage intensity, reaching maximal concentrations under total hail damage. Conversely, coutaric acid decreased progressively, with statistically significant differences observed across all treatments. Collectively, these findings indicate that hail damage significantly affects hydroxycinnamic acid metabolism in berry skins, selectively increasing compounds associated with antioxidant and defence mechanisms while reducing others, likely reflecting metabolic redirection. This differentiation underscores the complexity of grape physiological responses to mechanical stress. The findings demonstrate that hail induces significant and varied alterations in individual compounds, indicating redistribution of phenolic metabolism under mechanical stress.

### 2.6. Effect of Natural Hail Damage on Stilbenes and Phenolic Aldehyde

Notably, vanillin concentration rose significantly with increasing damage, showing the lowest levels in the control group and the highest in cases of total hail damage, while moderate damage resulted in intermediate concentrations (Table 6). Conversely, resveratrol did not show statistically significant differences between treatments, despite a slight numerical increase under hail conditions. The response of ε-viniferin was particularly marked, with significant and progressive increases correlating with damage intensity, suggesting activation of defence mechanisms linked to the biosynthesis of dimeric stilbenes. In contrast, piceid did not show statistically significant differences between treatments, although there was a tendency for an increase under total damage conditions. Overall, the results suggest that mechanical stress from hail selectively stimulates the synthesis of specific stilbenes and phenolic aldehydes in grape berry skins, potentially enhancing the antioxidant and defensive capacities of the skin tissues. The findings indicate that the extent of hail damage affects the metabolism of stilbenes and related phenolic compounds, with a general trend towards increased concentrations of certain compounds under greater stress.

### 2.7. Effect of Natural Hail Damage on Individual Flavonols and Flavones

The concentrations of individual flavonols and the flavone luteolin in berry skins under varying degrees of hail-induced mechanical stress are summarized in Table 7. The data reveal a differential response within these phenolic classes, indicating that the severity of hail damage significantly influences specific biosynthetic outcomes. Rutin showed a significantly lower concentration under conditions of total damage compared to both the control and moderate damage, between which there was no significant difference. In contrast, quercetin exhibited a marked and statistically significant increase under total damage, suggesting a possible activation of the flavonol biosynthetic pathway in response to severe stress. A similar trend was observed for luteolin, whose concentration increased significantly under total damage, while the control and moderate damage had lower and statistically similar values. Overall, these findings indicate that severe mechanical stress from hail induces a shift in the metabolism of flavonols and flavones, characterized by a decrease in certain glycosylated forms, such as rutin, and a simultaneous increase in aglycone or more bioactive forms, such as quercetin and luteolin. This metabolic adjustment may be associated with enhanced tissue antioxidant defence against hail-induced stress.

### 2.8. Principal Component Analysis (PCA)

Table 8 presents the pairwise correlation analysis between the variables examined, highlighting strong positive and negative correlations at a statistical significance level of *p* < 0.0001. Overall, the results indicate a close functional link between phenolic metabolism, antioxidant capacity, and ripening indices of the berries. Particularly strong positive correlations were observed between compounds of the hydroxycinnamic acid pathway and antioxidant activity. Specifically, caffeic acid was strongly correlated with caftaric and fertaric acids, as well as with antioxidant activity (FRAP and DPPH methods), while fertaric acid showed a positive correlation with the same measurements and a negative correlation with coutaric acid. The strong positive correlation with antioxidant activity (FRAP and DPPH methods) confirms the consistency of these two methods for assessing antioxidant capacity. Significant positive correlations were also recorded between individual phenolic categories. Flavones and flavonols were strongly correlated with total flavonoids and condensed tannins, while total phenolics were positively associated with these categories, confirming their contribution to the total phenolic load. In addition, strong positive correlations particularly with procyanidin B2 (and in the case of vanillic acid also with procyanidin B1) were shown, suggesting common metabolic pathways or coordinated biosynthesis. Regarding the ripening indices, the ripening index showed a strong positive correlation with °Brix and a strong negative correlation with titratable acidity, as expected. At the same time, gallic acid was positively correlated with proline, which may indicate coordinated physiological responses associated with stress conditions, including those induced by hail events. Conversely, negative correlations were mainly observed between compounds from different metabolic pathways. Sinapic acid showed strong negative correlations with caffeic acid, epicatechin, and caftaric acid, suggesting possible metabolic redistribution among individual branches of phenolic biosynthesis. Overall, the correlation analysis confirms that increased antioxidant activity and phenolic content are closely associated with changes in grape ripening and responses to stress conditions, such as hail, highlighting the multifactorial nature of grape physiological adaptation.

As anticipated, the ripening index showed a strong positive correlation with TSS and a strong negative correlation with titratable acidity (TA). Of particular interest is the positive correlation between gallic acid and proline (*p* < 0.0001). This relationship may reflect a joint physiological response associated with stress conditions such as hail-induced stress, wherein proline acts as an osmoprotectant and gallic acid serves as a precursor for protective tannins.

This synergistic response suggests the integration of nitrogen metabolism and phenylpropanoid pathway activation under stress adaptation, with proline and gallic acid contributing complementary roles to cellular protection and structural reinforcement.

To further clarify the relationships among the morphological, physicochemical, and phenolic parameters under different hail damage intensities, principal component analysis (PCA) was conducted. The first two principal components accounted for 85,6% of the total variance, with PC1 explaining 75.21% and PC2 contributing 10.43% (Table 9).

The first component (PC1) effectively distinguished variables associated with advanced ripening, phenolic accumulation, and antioxidant capacity from those related to acidity and specific hydroxycinnamic acids. The total soluble solids (°Brix) and the ripening index showed a strong positive correlation with PC1. Most phenolic classes—including flavan-3-ols, condensed tannins, flavonoids, and flavonols—as well as the stilbenic compounds (resveratrol, ε-viniferin, and piceid) and antioxidant indicators (FRAP, DPPH) were also positively correlated on this axis.

This clustering confirms a robust, coordinated response in which increased mechanical stress levels are intrinsically linked to both accelerated ripening indices and enhanced synthesis of secondary metabolites for antioxidant defence. In contrast, titratable acidity, sinapic acid, and, to a lesser extent, *p*-coumaric acid, showed a negative correlation with PC1, reinforcing the inverse relationship between these parameters and the overall phenolic content.

**Figure 1:**
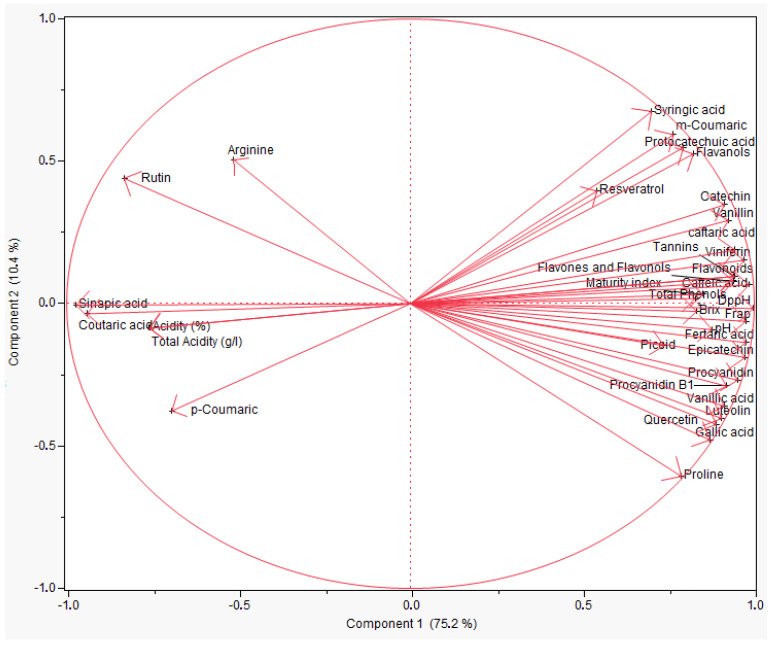
Principal component analysis (PCA) plot showing the distribution of variables along the first two principal components (PC1 and PC2), which together explain 85.6% of the total variance. Variables are represented as vectors (arrows); their direction indicates correlation (positive for similar directions, negative for opposite directions), and their length reflects their contribution to the components.

Principal component analysis (PCA) was used to investigate the correlations between the morphological, physicochemical, and phenolic parameters of grapes subjected to different levels of natural hail damage. PC1 primarily separates variables associated with increased ripening, phenolic content, and antioxidant activity from those linked to higher acidity, particularly hydroxycinnamic acids. Positive correlations with PC1 are observed for °Brix, the ripening index, as well as most phenolic compounds (flavanols, tannins, catechin, procyanidins, flavonoids, flavones/flavonols), together with antioxidant capacity indicators (FRAP, DPPH) and compounds such as resveratrol, ε-viniferin and piceid. The alignment of these parameters in the same direction indicates a strong positive correlation between phenolic metabolism, ripening, and antioxidant defence under intense stress conditions. In contrast, titratable acidity, sinapic acid, coutaric acid and *p*-coumaric acid show a positive correlation with PC1 (Figure 1), indicating an inverse relationship with the degree of ripening and phenolic accumulation. *p*-Coumaric acid also diverges markedly in the negative direction of PC2, suggesting a distinct metabolic behaviour compared to other hydroxycinnamic derivatives. PC2 primarily separated individual amino acids and phenolic subcategories, with arginine and rutin associated with positive correlation (Figure 1), while certain phenolics (such as piceid and fertaric acid) were associated with negative correlation. This distribution indicates a differentiation between metabolic responses linked to the nitrogen status of the must and those related to structural or phenolic adaptations of the skin.

Overall, PCA confirms that increased hail damage is associated with enhanced ripening, greater accumulation of phenolic compounds, and higher antioxidant activity, alongside a decrease in acidity and a redistribution of individual hydroxycinnamic derivatives. These findings highlight the complex physiological and metabolic responses of grapes to mechanical stress.

## 3. Discussion

The present study demonstrates that natural hail induces a highly coordinated shift in the must composition of ‘Thompson seedless’ grapes, particularly in the metabolic profile of skin phenolics. Although the primary ripening indices (TSS, TA, and maturity index) remained statistically stable, substantial variations in pH, proline concentration, and antioxidant capacity indicate sophisticated physiological adaptation to acute mechanical stress, similar to responses observed in other abiotic stress models [11,17]. Nevertheless, the relative stability of the sugar–acid balance indicates that the core carbon allocation processes associated with berry ripening were not substantially disrupted. The significant elevation of pH under severe damage likely reflects localized shifts in organic acid compartmentalisation or compromised vacuolar membrane integrity [12]. A key finding of this study is the significant accumulation of proline under total damage conditions. Proline serves as a hallmark indicator of abiotic stress, functioning in osmoregulation, protein stabilization, and redox homeostasis maintenance [15,16].

Proline accumulation indicates activation of defence mechanisms against mechanical injury. Conversely, arginine exhibited no significant variation, suggesting selective regulation of nitrogen metabolism. The most pronounced effect of hailstorms concerned phenolic metabolism. A progressive increase in total phenols, flavonoids, flavanols, tannins, flavones and flavonols, and antioxidant capacity (FRAP and DPPH) was observed with escalating damage intensity. The strong correlation between the two antioxidant assessment methods confirms that enhanced antioxidant capacity is directly linked to phenolic compound accumulation. Activation of the phenylpropanoid pathway under stress conditions is a widely documented phenomenon, leading to elevated biosynthesis of antioxidants and structural phenolics [9,10]. Similar increases have been documented under water deficit, ultraviolet radiation, and heat stress [17,18].

At the individual compound level, (+)-catechin, (−)-epicatechin, and procyanidins showed progressive increases. These flavan-3-ols play a dual protective role by scavenging reactive oxygen species (ROS) and reinforcing cell wall structure through tannin–protein and tannin–polysaccharide interactions [19]. In addition, the divergent behaviour of hydroxycinnamic acids—specifically, the increase in caffeic, caftaric, and fertaric acids alongside the decrease in sinapic and coutaric acids—suggests a redistribution of metabolic flux, prioritizing key antioxidant compounds and precursors involved in wound response [10,20].

Furthermore, stilbene metabolism was significantly modulated, with a marked increase in ε-viniferin under severe damage. The accumulation of this resveratrol dehydrodimer, a potent phytoalexin, suggests a shift toward stilbene oligomerization to enhance antioxidant and antimicrobial defence [14,21]. The distinction between the glycoside rutin and its aglycones (quercetin and luteolin) further supports a shift toward more bioactive, ROS-scavenging forms [22].

Correlation analysis and principal component analysis (PCA) further integrated these findings, revealing a clear separation between phenolic accumulation patterns and acidity-related parameters, highlighting the coordinated nature of the grapevine metabolic response.

Overall, these results indicate that acute hail events act as a strong abiotic stressor, activating the phenylpropanoid pathway and reorganizing secondary metabolism. While primary ripening parameters remained largely unaffected, the biochemical modifications in berry skin composition may significantly influence the organoleptic and functional properties of grapes and derived products [4,5], underscoring the adaptive plasticity of grapevine responses to mechanical stress.

## 4. Materials and Methods

### 4.1. Vineyard Conditions and Grape Sampling

The trial was conducted in a commercial table-grape vineyard of *Vitis vinifera* L. cv. Thompson seedless located in Laliotis, a municipal unit of Kiato (northeastern Peloponnese, Greece), during the 2015 growing season. All information about the vineyard is reported in the article previously published by Petoumenou et al. [3]. According to this article, a 20 min hailstorm took place on 18 June 2015 (the 169th day of the year, DOY), four weeks after full bloom (known as development stage EL-33 [23]); it was preceded by heavy rain (36 mm/h) and accompanied by wind velocities up to 78 km/h and hailstone diameters of 25–30 mm. The hailstorm hit one part of the vineyard, causing moderate damage to the grapes, and another part, where it caused almost total damage. So, three treatments were compared: control vines (ND, hail-damaged); medium-hail-damaged (MHD) vines, the canopy of which was visibly damaged by the hailstorm; and total-hail-damaged vines. Grapes were selected, chosen on the basis of being the most representative in each treatment. Grapes were randomly selected from different vines for each treatment to analyze their polyphenolic profiles. They were collected from the main shoots at various positions. Each treatment was replicated three times, and each replicate consisted of five grapes.

### 4.2. Fruit Composition

At harvest, performed on 5 September 2015 (DOY 248), when the sugar accumulation on ND vines reached 20 °Brix, grapes were randomly selected from each of the treatments. The sampling process involved the random selection of five (5) grape clusters from different vines of each treatment and three (3) sampling processes. The clusters were collected from main shoots positioned randomly on the vine. Each sampling counted as one (1) replication. To separate the skins from their respective berries, samples from each replication were prepared for spectrophotometric and HPLC analyses.

#### 4.2.1. Reagents and Chemicals

The various polyphenolic compounds analyzed were identified according to their order of elution and the retention times of the pure compounds. Non-coloured polyphenolics were purchased from several different suppliers. Specifically, gallic acid, protocatechuic acid, catechin, vanillic acid, caffeic acid, syringic acid, vanillin, epicatechin, ferulic acid, sinapic acid, *m*-coumaric acid, *p*-coumaric acid, and rutin were purchased from Sigma (St. Louis, MO, USA); luteolin, procyanidin B1, procyanidin B2, ε-viniferin, quercetin, trans-resveratrol, and piceid were purchased from Extrasynthese (Genay, France); and coutaric acid, caftaric acid, and fertaric acid were purchased from PhytoLab GmbH & Co. KG (Vestenbergsgreuth, Germany).

#### 4.2.2. Determination of Total Soluble Solids, pH, Titratable Acidity, and Maturity Index

The total soluble solids (TSS) in the must were determined using an ATAGO N1 (ATAGO Co., Ltd., Tokyo, Japan)—a refractometer with a 0–32 °Brix measurement range and 0.28 °Brix increments; temperature compensation was not applied. Total titratable acidity (TA) was measured by titration with 0.1 N NaOH and expressed as tartaric acid, the predominant organic acid in *Vitis vinifera* L. grapes. The maturity index was calculated as the ratio of total soluble solids (TSS) to titratable acidity (TA).

#### 4.2.3. Sample Preparation for UV Spectrophotometric and HPLC Analysis

For each replication, approximately 100 berries were manually peeled to isolate the skins. The skins were lyophilized and subsequently pulverized in a mill to produce a fine powder. These samples were stored at −80 °C prior to extraction. Berry skin extracts were prepared by weighing 0.4 g of the dried powder into 4 mL of an extraction solvent (water/methanol/acetone/HCl; 19:40:40:1 *v*/*v*/*v*/*v*). The mixture was homogenized for 1 min at 8000 rpm (revolutions per minute) using an Ultra-Turrax homogenizer (IKA-Werke GmbH & Co. KG, Staufen, Germany). Following homogenization, the extracts were agitated in a vacuum incubator at 150 rpm for 30 min at a controlled temperature of 25 °C. The samples were then centrifuged at 4500 rpm for 10 min. The supernatants were collected, and the extraction procedure was performed in triplicate. All fractions were pooled and stored at −80 °C pending further analysis.

### 4.3. Determination of Total Phenols

The total phenolic content was determined according to the methods described by [24,25]. An aliquot of the extract was dissolved in methanol 1:10. To measure the total phenols, 0.5 mL of the dissolved extract was added to 10 mL HCL 1 M. The tubes stayed in the dark for 3 h and the concentration of total phenols was determined spectrophotometrically at 280 nm. The concentration of total phenols was estimated from a calibration curve, constructed by plotting known solutions of catechin (12.5–200 μg/mL). The results are expressed as means (milligrams of catechin equivalent per gram of fresh matter). All spectrophotometric measurements were performed in triplicate to ensure analytical precision.

### 4.4. Determination of Total Flavonoid Content

Total flavonoid content (TFC) was determined via the colorimetric method described by [26]. An aliquot of the extract was dissolved in methanol 1:10. Briefly, 0.25 mL of the dissolved extract or catechin standard solution was mixed with 1.25 mL of distilled water in a test tube followed by addition of 75 μL of 5% NaNO_2_ solution. After 6 min, 150 μL of a 10% AlCl_3_·6H_2_O solution was added and allowed to stand for another 5 min, and 0.5 mL of 1 mL NaOH was added. The mixture was brought to 2.5 mL with distilled water and mixed well. Absorbance was measured immediately against the blank at 510 nm. The concentration of total flavanols was estimated from a calibration curve, constructed by plotting known solutions of catechin (12.5–200 μg/mL). The results are expressed as mean (milligrams of catechin equivalent per gram of fresh matter).

### 4.5. Determination of Total Flavanol Content

The total flavanol content was determined using the p-dimethylaminocinnamaldehyde (DMACA) colorimetric method, following the procedures established by [27,28]. The extract (0.2 mL), diluted 1:50 with methanol, was introduced into a 15 mL tube and 1 mL DMACA solution (0.1% in 1 N HCl in MeOH) was added. The mixture was vortexed and allowed to react at room temperature for 10 min. Following this, the absorbance at 640 nm was read against the blank. The concentration of total flavanols was estimated from a calibration curve, constructed by plotting known solutions of catechin (12.5–200 μg/mL). The results are expressed as mean (milligrams of catechin equivalent per gram of fresh matter).

### 4.6. Determination of Total Flavones and Flavonols

The total flavone and flavonol content was determined according to the spectrophotometric method described by [29] and adapted by [30]. An aliquot (2 mL) of the test solution, 20 mL methanol and 1 mL 5% aluminum chloride in methanol (*w*/*v*) were mixed in a volumetric flask and the volume was made up to 50 mL with methanol. The mixture was left for 30 min and the absorbance was measured at 425 nm. Quantification was performed using a calibration curve generated from rutin standards within a concentration range of 12.5–200 μg/mL. Absorbance was recorded at 360 nm (the characteristic maximum for these phenolic classes). Results are expressed as milligrams of rutin per gram of fresh skin (mg rutin/g skin).

### 4.7. Determination of Total (Condensed) Tannins

Total condensed tannin content was determined using the methyl cellulose precipitation (MCP) assay, according to the method described by [31], with minor modifications. Treatment sample: Six hundred microliters of methyl cellulose solution (0.04%) was added to each sample (50 μL) in a 2 mL centrifuge tube which was inverted several times and allowed to stand for 2–3 min at room temperature. Then, 400 μL of saturated ammonium sulphate solution was added and the volume was made up with water to 2 mL. The solution was incubated at room temperature for 10 min and then centrifuged for 15 min at 10,000× *g*. The absorbance was measured at 280 nm.

Control sample: In a 2 mL centrifuge tube, 400 μL of saturated ammonium sulphate solution was added to 30 μL of each sample and the volume was made up with deionized water and mixed by tube inversion. The solution was allowed to stand at room temperature for 10 min and then centrifuged for 15 min at 10,000× *g*. Deionized water was used instead of methyl cellulose solution. The absorbance was measured at 280 nm.

Quantification: The condensed tannin concentration is determined by subtracting the A280 of the methyl cellulose-treated sample from the A280 of the control sample. The concentration of condensed tannins was quantified via a calibration curve generated from catechin standards within a range of 12.5–200 μg/mL. Absorbance was recorded at 280 nm, representing the difference between the control (non-precipitated) and the precipitated samples. Results are expressed as milligrams of catechin per gram of fresh skin (mg catechin/g skin).

### 4.8. HPLC Analysis of Polyphenols

The profiling and quantification of polyphenols were conducted via high-performance liquid chromatography (HPLC). The analytical system consisted of a Nexera X2 gradient pump (Shimadzu Corporation, Kyoto, Japan), a ProStar Model 410 autosampler, and a ProStar Model 330 Photodiode Array Detector (PDA) (Varian Inc., Palo Alto, CA, USA). Separations were performed on a reversed-phase XSelect C18 column (250 mm × 4.6 mm, 5 μm; Waters Corporation, Milford, MA, USA) maintained at a constant temperature of 25 °C. Identification of phenolic compounds was achieved by comparing retention times and UV-Vis spectral data against authentic external standards.

### 4.9. Determination of Antioxidant Activity (DPPH Radical Scavenging Assay)

The free-radical scavenging activity was evaluated using the DPPH (2,2-diphenyl-1-picrylhydrazyl) colorimetric assay, according to the method of [32] with minor modifications. Antioxidant capacity was determined using the DPPH radical scavenging assay. A calibration curve was prepared using Trolox standard solutions. Briefly, 12.5 mg of dry Trolox were dissolved in a 25 mL volumetric flask and brought to volume with absolute ethanol, yielding a 2 mM stock solution. Aliquots of the stock solution (0, 0.04, 0.08, 0.10, 0.20, 0.30, 0.40, 0.50, 0.625, 0.75, 0.875, and 1.00 mL) were transferred into 1 mL tubes. Subsequently, 50 μL of each standard solution was mixed with 1.950 μL of DPPH solution (3.68 × 10^−4^ M), vortexed, and incubated in the dark for 30 min. Absorbance was measured at 515 nm using a spectrophotometer. For each replicate, three spectrophotometric measurements were performed and the mean value was used for further calculations The calibration curve was constructed by plotting absorbance against Trolox concentration, and sample antioxidant capacity was expressed as Trolox equivalents based on the resulting regression equation. The scavenging capacity was quantified using 6-hydroxy-2,5,7,8-tetramethylchroman-2-carboxylic acid (Trolox) as the external standard. A linear calibration curve was established using Trolox concentrations ranging from 2.0 to 1598.1 μM. Absorbance was recorded at 515 nm (the maximum absorbance of the DPPH radical) until the reaction reached a steady state. Results are expressed as milligrams of Trolox per gram of fresh skin (mg TE/g skin).

### 4.10. Determination of Ferric Reducing Antioxidant Power (FRAP)

The total reducing capacity of the berry skin extracts was determined using the Ferric Reducing Antioxidant Power (FRAP) assay, according to the methodology of [33]. The assay is based on the reduction of a ferric-tripyridyltriazine (Fe^3+^-TPTZ) complex to its ferrous (Fe^2+^) form at low pH, resulting in an intense blue-coloured complex measured spectrophotometrically. The FRAP working reagent was freshly prepared daily by mixing 25 mL of 0.3 M acetate buffer (pH 3.6), 2.5 mL of 10 mM TPTZ solution prepared in 40 mM HCl, and 2.5 mL of 20 mM FeCl_3_·6H_2_O solution. The reagent was preheated to 37 °C prior to use. Grape extracts were diluted 1:80 with HPLC-grade methanol before analysis. Subsequently, 0.1 mL of diluted extract was mixed with 1.1 mL of FRAP reagent and incubated in the dark at 37 °C for 10 min. Absorbance was then measured at 593 nm using a spectrophotometer. A blank solution was prepared by replacing the extract with HPLC-grade methanol and following the same procedure. For each replicate, three spectrophotometric measurements were performed, and the mean value was used for further calculations. Quantification was achieved using a calibration curve constructed with Trolox standard solutions of known concentrations, and the antioxidant capacity of the samples was expressed as Trolox equivalents. Quantification was performed using a calibration curve generated from Trolox standards. Absorbance was recorded at 593 nm after an incubation time of 10 min at 37 °C. The results are expressed as milligrams of Trolox per gram of fresh skin (mg TE/g skin).

### 4.11. HPLC Analysis of Individual Polyphenols

In order to measure individual polyphenols with HPLC, liquid extraction was carried out. An extract of 0.5 mL was mixed up with 4 mL ethyl acetate with a vortex, and the supernatant was separated. The supernatant was then washed twice with distilled water. Next, the supernatants were evaporated with a sample concentrator, and the pellets were dissolved in 1 mL of 50% methanol in water. Last, prior to the HPLC analysis, 1.5 mL was filtered through a 0.22 μm membrane. The following were determined using the HPLC PDA system (Shimadzu Nexera): monomeric and dimeric polyphenols (+)-catechin, (•)-epicatechin, proanthocyanidins B1 and B2, gallic acid, protocatechuic acid, caftaric acid, vanillic acid, caffeic acid, coutaric acid, vanillin, syringic acid, fertaric acid, *p*-coumaric, *m*-coumaric, piceid, ferulic acid, sinapic acid, rutin, trans-resveratrol, ε-viniferin, quercetin, and luteolin. The separation of monomeric and dimeric polyphenols was performed using a reversed-phase XSelect C18 column (250 × 4.6 mm i.d., 5 μm; Waters Corporation, Milford, MA, USA), maintained at a constant temperature of 25 °C. The eluent was composed of (a) H_2_O/HClO_4_ (99:1) and (b) CH_3_OH (100). The flow rate was 0.5 mL/min. The following linear gradient programme was used for the elution: 0% B for 2 min; from 0 to 5% B in 16 min; from 5 to 10% B in 25 min; from 10 to 15% B in 50 min; from 15 to 25% B in 90 min; from 25 to 45% B in 120 min; from 45 to 75% B in 145 min; from 75 to 90% B in 150 min; and from 90 to 95% B in 155 min, followed by a return to the initial conditions in ten (10) minutes and re-equilibration of the column. The chromatogram was monitored at 280, 320, and 360 nm. The column was returned to initial conditions over 10 min and re-equilibrated before the next injection. The eluate was monitored via Photodiode Array (PDA) at 280 nm (phenolic acids and flavan-3-ols), 320 nm (hydroxycinnamic acids and stilbenes), and 360 nm (flavonols).

### 4.12. Statistical Analysis

All data are presented as mean ± standard error (SE) of three replicates. Each replicate consisted of a composite sample of three grapes. All analytical determinations were performed in triplicate (technical replicates). The data were subjected to one-way analysis of variance (ANOVA) to determine the significant differences among the treatments. Mean separation was performed using Tukey’s Honestly Significant Difference (HSD) test with a significance threshold of *p* < 0.05. To explore the relationships between the measured variables and their contribution to total variability, principal component analysis (PCA) was conducted. All statistical procedures, including correlation analyses, were performed using JMP v.10 software (SAS Institute Inc., Cary, NC, USA).

## 5. Conclusions

This study investigated the influence of natural hail damage on the physicochemical parameters of must, grape skin phenolic composition, and antioxidant capacity. The results showed that phenolic compound concentrations were significantly affected by natural hail, with higher concentrations observed under more severe damage conditions. These findings suggest the activation of defence mechanisms and metabolic adaptations in response to mechanical stress, with direct implications for grape quality and potential wine styles. In conclusion, understanding the adaptive biochemical responses of grapevines to climatic stressors is crucial for improving vineyard management practices and predicting grape and wine quality, given the increasing frequency and intensity of extreme weather phenomena. This knowledge is essential for agronomists and winemakers to anticipate and manage the qualitative consequences of such climatic stressors, thereby ensuring the resilience and sustainability of grape production and wine quality under changing environmental conditions.

## Figures and Tables

**Table 1:** Effects of natural hail on fruit composition.

Parameter	Control	Medium Hail Damage	Total Hail Damage
TTS (°Brix)	20.03 ± 1.13 ^a^	21.00 ± 0.85 ^a^	23.43 ± 0.27 ^a^
pH	4.27 ± 0.08 ^b^	4.37 ± 0.09 ^ab^	4.76 ± 0.10 ^a^
Titratable Acidity (TA, g/L)	3.93 ± 0.13 ^a^	3.73 ± 0.24 ^a^	3.42 ± 0.07 ^a^
Maturity index (TSS/TA)	5.11 ± 0.398 ^a^	5.70 ± 0.561 ^a^	6.85 ± 0.213 ^a^
Proline (mg/mL)	0.299 ± 0.003 ^b^	0.228 ± 0.008 ^c^	0.502 ± 0.002 ^a^
Arginine (mg/mL)	0.640 ± 0.031 ^a^	0.658 ± 0.029 ^a^	0.578 ± 0.012 ^a^

Mean values (Mean ± SE) in the same row assigned different letters are significantly different according to Tukey’s range test at *p* ≤ 0.05.

**Table 2:** Effects of natural hail on the phenolic berry skin profile.

Compound	Control	Medium Hail Damage	Total Hail Damage
Flavanols (mg catechin/g fresh weight (F.W.))	9.12 ± 0.10 ^b^	11.81 ± 0.01 ^a^	11.99 ± 0.02 ^a^
Tannins (mg catechin/g F.W.)	1.30 ± 0.03 ^b^	1.40 ± 0.03 ^ab^	1.53 ± 0.02 ^a^
Flavonoids (mg catechin/g F.W.)	0.390 ± 0.010 ^b^	0.422 ± 0.009 ^ab^	0.458 ± 0.005 ^a^
Flavones and Flavonols (mg rutin/g F.W.)	0.117 ± 0.003 ^b^	0.126 ± 0.003 ^b^	0.137 ± 0.002 ^a^
Total Phenols (mg gallic acid/g F.W.)	0.035 ± 0.001 ^b^	0.038 ± 0.001 ^ab^	0.041 ± 0.002 ^a^
Frap (mg trolox/g F.W.)	14.98 ± 0.146 ^c^	17.13 ± 0.324 ^b^	20.83 ± 0.134 ^a^
DppH (mg trolox/g F.W.)	7.33 ± 0.133 ^c^	10.48 ± 0.379 ^b^	15.55 ± 0.044 ^a^

Mean values (Mean ± SE) in the same row assigned different letters are significantly different according to Tukey’s range test at *p* ≤ 0.05.

**Table 3:** Effects of natural hail on berry skin individual flavanols (μg/g F.W.).

Compound	Control	Medium Hail Damage	Total Hail Damage
Catechin	1421.99 ± 10.33 ^c^	1847.00 ± 7.38 ^b^	2022.89 ± 62.66 ^a^
Epicatechin	143.87 ± 0.88 ^c^	162.45 ± 1.16 ^b^	222.13 ± 4.57 ^a^
Procyanidin B1	1599.30 ± 8.060 ^b^	1624.07 ± 27.76 ^b^	1792.34 ± 14.81 ^a^
Procyanidin B2	296.22 ± 5.70 ^b^	312.02 ± 6.02 ^b^	406.82 ± 0.69 ^a^

Mean values (Mean ± SE) in the same row assigned different letters are significantly different according to Tukey’s range test at *p* ≤ 0.05.

**Table 4:** Effects of natural hail on berry skin hydroxybenzoic acids (μg/gr F.W.).

Compound	Control	Medium Hail Damage	Total Hail Damage
Protocatechuic acid	12.34 ± 0.074 ^b^	18.56 ± 0.89 ^a^	18.68 ± 0.263 ^a^
Gallic acid	7.83 ± 0.12 ^b^	7.43 ± 0.05 ^b^	12.24 ± 0.28 ^a^
Vanillic acid	1.54 ± 0.023 ^b^	1.65 ± 0.068 ^b^	3.78 ± 0.231 ^a^
Syringic acid	15.28 ± 0.283 ^c^	32.88 ± 0.504 ^a^	30.29 ± 0.153 ^b^

Mean values (Mean ± SE) in the same row assigned different letters are significantly different according to Tukey’s range test at *p* ≤ 0.05.

**Table 5:** Effects of natural hail on berry skin hydroxycinnamic acids and their respective tartaric acid esters (μg/g F.W.).

Compound	Control	Medium Hail Damage	Total Hail Damage
Sinapic acid	66.72 ± 0.386 ^a^	45.51 ± 7.497 ^b^	14.27 ± 0.814 ^c^
Caffeic acid	1.41 ± 0.021 ^c^	2.65 ± 0.021 ^b^	4.06 ± 0.031 ^a^
*p*-Coumaric acid	12.86 ± 0.42 ^a^	9.22 ± 2.34 ^a^	8.23 ± 0.19 ^a^
*m*-Coumaric acid	0.349 ± 0.011 ^b^	2.24 ± 0.061 ^a^	2.17 ± 0.028 ^a^
Caftaric acid	1229.04 ± 4.76 ^c^	1376.89 ± 21.74 ^b^	1496.95 ± 25.33 ^a^
Coutaric acid	197.82 ± 2.75 ^a^	177.46 ± 3.59 ^b^	151.17 ± 4.83 ^c^
Fertaric acid	412.89 ± 4.32 ^c^	479.26 ± 13.56 ^b^	639.02 ± 4.88 ^a^

Mean values (Mean ± SE) in the same row assigned different letters are significantly different according to Tukey’s range test at *p* ≤ 0.05.

**Table 6:** Effects of natural hail on berry skin stilbenes and phenolic aldehyde (μg/g skin F.W.).

	Compound	Control	Medium Hail Damage	Total Hail Damage
Phenolic aldehyde	Vanillin	2.33 ± 0.087 ^c^	4.31 ± 0.104 ^ab^	5.31 ± 0.261 ^a^
Individual Stilbenes	Resveratrol	8.60 ± 0.87 ^a^	9.66 ± 0.52 ^a^	9.82 ± 0.77 ^a^
	ε-Viniferin	1.03 ± 0.032 ^c^	7.52 ± 0.255 ^b^	12.72 ± 0.286 ^a^
	Piceid	223.16 ± 2.38 ^a^	232.04 ± 2.37 ^a^	250.71 ± 11.89 ^a^

Mean values (Mean ± SE) in the same row assigned different letters are significantly different according to Tukey’s range test at *p* ≤ 0.05.

**Table 7:** Effects of natural hail on berry skin individual flavonols and flavone (μg/g F.W.).

	Compound	Control	Medium Hail Damage	Total Hail Damage
Individual Flavonols	Rutin	7114.4 ± 86.0 ^a^	7359.5 ± 218.9 ^a^	5567.8 ± 173.2 ^b^
	Quercetin	6.22 ± 0.080 ^b^	5.95 ± 0.683 ^b^	17.29 ± 0.311 ^a^
Individual Flavones	Luteolin	2275 ± 0.073 ^b^	1.931 ± 0.437 ^b^	17.78 ± 0.677 ^a^

Mean values (Mean ± SE) in the same row assigned different letters are significantly different according to Tukey’s range test at *p* ≤ 0.05.

**Table 8:** Paired comparison analysis of the variables evaluated.

Compound	Strong Positive Correlations (*p* < 0.0001)	Strong Negative Correlations (*p* < 0.0001)
Caffeic acid	Caftaric acid, Fertaric acid, Frap, DppH	
DppH	Frap	
Fertaric acid	Frap, DppH	Coutaric acid
Flavones and Flavonols	Flavonoids, Tannins	
Flavonoids	Tannins	
Gallic acid	Proline	
Luteolin	Vanillic acid, Procyanidin B2, Quercetin	
Maturity index	TSS	Titratable acidity (g/L)
*m*-Coumaric	Flavanols	
Procyanidin	Gallic acid	
Quercetin	Procyanidin B2, Vanillic acid	
Sinapic acid		Caffeic acid, Epicatechin, Caftaric acid
Total Phenols	Flavonoids, Tannins, Flavones and Flavonols	
Vanillic acid	Procyanidin B2, Procyanidin B1	
Vanillin	Caffeic acid, Catechin, Caftaric acid	
Viniferin	Caftaric acid, Catechin, Vanillin, Caffeic acid	

**Table 9:** Principal components (PCs) of the variables evaluated.

**Principal components**			
1	2	3	4
**Contribution to variability (%)**			
75.21	10.43	6.09	3.74
**Eigenvalue**			
27.07	3.75	2.19	1.34
**Variables**			
Frap	Quercetin	TSS	
DppH	Vanillic acid	pH	Arginine
caftaric acid	Proline	Titratable acidity	Tannins
Fertaric acid	Flavanols	Maturity index	Flavonoids
Vanillin	Gallic acid	Acidity	Flavones and Flavonols
Caffeic acid	Protocatechuic acid		Total Phenols
Epicatechin	Catechin		Coutaric acid
Sinapic acid	Syringic acid		*p*-Coumaric
Viniferin	Luteolin		Piceid
Procyanidin B1	*m*-Coumaric		Resveratrol
Procyanidin B2	Rutin		

## Data Availability

The original contributions presented in this study are included in the article. Further inquiries can be directed to the corresponding authors.

## References

[1] Staurakas D.E. (2015). Ampelography.

[2] Petoumenou D.G., Liava V. (2025). Sustainable Foliar Applications to Improve Grapevine Responses to Drought, High Temperatures, and Salinity: Impacts on Physiology, Yields, and Berry Quality. Plants.

[3] Petoumenou D.G., Biniari K., Xyrafis E., Mavronasios D., Daskalakis I., Palliotti A. (2019). Effects of Natural Hail on the Growth, Physiological Characteristics, Yield, and Quality of *Vitis vinifera* L. cv. Thompson Seedless under Mediterranean Growing Conditions. Agronomy.

[4] Waterhouse A.L. (2002). Wine Phenolics. Ann. N. Y. Acad. Sci..

[5] Jackson R.S. (2020). Wine Science: Principles and Applications.

[6] Teixeira A., Eiras-Dias J., Castellarin S.D., Gerós H. (2013). Berry Phenolics of Grapevine under Challenging Environments. Int. J. Mol. Sci..

[7] Ramos M.C., Ibáñez Jara M.Á., Rosillo L., Salinas M.R. (2024). Effect of Temperature and Water Availability on Grape Phenolic Compounds and Their Extractability in Merlot Grown in a Warm Area. Sci. Hortic..

[8] Küskü D.Y. (2026). Abiotic Stress Effects on Phenolic Metabolism in *Vitis vinifera*: Implications for Grape Quality. J. Plant Growth Regul..

[9] Dixon R.A., Paiva N.L. (1995). Stress-Induced Phenylpropanoid Metabolism. Plant Cell.

[10] Vogt T. (2010). Phenylpropanoid Biosynthesis. Mol. Plant.

[11] Cramer G.R., Urano K., Delrot S., Pezzotti M., Shinozaki K. (2011). Effects of Abiotic Stress on Plants: A Systems Biology Perspective. BMC Plant Biol..

[12] Keller M. (2015). The Science of Grapevines: Anatomy and Physiology.

[13] Rice-Evans C.A., Miller N.J., Paganga G. (1997). Antioxidant Properties of Phenolic Compounds. Trends Plant Sci..

[14] Jeandet P., Clément C., Courot E. (2014). Resveratrol Production at Large Scale Using Plant Cell Suspensions. Eng. Life Sci..

[15] Szabados L., Savouré A. (2010). Proline: A Multifunctional Amino Acid. Trends Plant Sci..

[16] Kavi Kishor P.B., Sreenivasulu N. (2014). Is Proline Accumulation per se Correlated with Stress Tolerance or Is Proline Homeostasis a More Critical Issue?. Plant Cell Environ..

[17] Castellarin S.D., Pfeiffer A., Sivilotti P., Degan M., Peterlunger E., Di Gaspero G. (2007). Transcriptional Regulation of Anthocyanin Biosynthesis in Ripening Fruits of Grapevine under Seasonal Water Deficit. Plant Cell Environ..

[18] Downey M.O., Dokoozlian N.K., Krstic M.P. (2006). Cultural Practice and Environmental Impacts on the Flavonoid Composition of Grapes and Wine: A Review of Recent Research. Am. J. Enol. Vitic..

[19] Cheynier V., Dueñas-Paton M., Salas E., Maury C., Souquet J.M., Sarni-Manchado P., Fulcrand H. (2006). Structure and Properties of Wine Pigments and Tannins. Am. J. Enol. Vitic..

[20] Mattivi F., Guzzon R., Vrhovsek U., Stefanini M., Velasco R. (2006). Metabolite Profiling of Grape: Flavonols and Anthocyanins. J. Agric. Food Chem..

[21] Pezet R., Viret O., Gindro K., Hemantaranjan A. (2004). Plant–Microbe Interaction: The *Botrytis* Grey Mould of Grapes. Advances in Plant Physiology, Biology, Biochemistry, Epidemiology and Control Management.

[22] Agati G., Brunetti C., Fini A., Gori A., Guidi L., Landi M., Sebastiani F., Tattini M. (2020). Are Flavonoids Effective Antioxidants in Plants? Twenty Years of Our Investigation. Antioxidants.

[23] Lorentz D.H., Eichhorn K.W., Bleiholder H., Klose R., Meier U., Weber E. (1995). Growth Stages of the Grapevine: Phenological Growth Stages of the Grapevine (*Vitis vinifera* L. ssp. *vinifera*)—Codes and Descriptions According to the Extended BBCH Scale. Aust. J. Grape Wine Res..

[24] Somers T.C., Evans M.E. (1977). Spectral Evaluation of Young Red Wines: Anthocyanin Equilibria, Total Phenolics, Free and Molecular SO_2_, Chemical Age. J. Sci. Food Agric..

[25] Biniari K., Gerogiannis O., Daskalakis I., Bouza D., Stavrakaki M. (2018). Study of Some Qualitative and Quantitative Characters of the Grapes of Indigenous Greek Grapevine Varieties (*Vitis vinifera* L.) Using HPLC and Spectrophotometric Analyses. Not. Bot. Horti Agrobot. Cluj-Napoca.

[26] Dewanto V., Wu X., Adom K.K., Liu R.H. (2002). Thermal Processing Enhances the Nutritional Value of Tomatoes by Increasing Total Antioxidant Activity. J. Agric. Food Chem..

[27] Vivas N., Glories Y., Lagune L., Saucier C., Augustin M. (1994). Estimation du Degré de Polymérisation des Procyanidines du Raisin et du Vin par la Méthode au *p*-Dimethylaminocinnamaldéhyde. J. Int. Sci. Vigne Vin.

[28] McMurrough I., Madigan D., Donnelly D., Hurley J., Doyle A.M., Hennigan G., McNulty N., Smyth M.R. (1996). Control of Ferulic Acid and 4-Vinyl Guaiacol in Brewing. J. Inst. Brew..

[29] Bonvehí J.S., Gutiérrez A.L. (2011). Antioxidant Activity and Total Phenolics of Propolis from the Basque Country (Northeastern Spain). J. Am. Oil Chem. Soc..

[30] Biniari K., Gerogiannis O., Daskalakis I., Bouza D., Stavrakaki M. (2020). Polyphenolic Compounds and Antioxidants of Skin and Berry Grapes of Greek *Vitis vinifera* Cultivars in Relation to Climate Conditions. Food Chem..

[31] Sarneckis C.J., Dambergs R.G., Jones P., Mercurio M., Herderich M.J., Smith P.A. (2006). Quantification of Condensed Tannins by Precipitation with Methyl Cellulose: Development and Validation of an Optimised Tool for Grape and Wine Analysis. Aust. J. Grape Wine Res..

[32] Brand-Williams W., Cuvelier M.E., Berset C. (1995). Use of a Free Radical Method to Evaluate Antioxidant Activity. LWT—Food Sci. Technol..

[33] Benzie I.F.F., Strain J.J. (1996). The Ferric Reducing Ability of Plasma (FRAP) as a Measure of Antioxidant Power: The FRAP Assay. Anal. Biochem..

